# A polynomial delay algorithm for the enumeration of bubbles with length constraints in directed graphs

**DOI:** 10.1186/s13015-015-0046-4

**Published:** 2015-06-27

**Authors:** Gustavo Sacomoto, Vincent Lacroix, Marie-France Sagot

**Affiliations:** INRIA Rhône-Alpes, 38330 Montbonnot Saint-Martin, France; Université de Lyon, 69000 Lyon, France; Université Lyon 1, Lyon, France; CNRS, UMR5558, Laboratoire de Biométrie et Biologie Evolutive, 69622 Villeurbanne, France

**Keywords:** Alternative splicing, RNA-seq, De Bruijn graphs, Bubbles, Enumeration algorithms

## Abstract

**Background:**

The problem of enumerating bubbles with length constraints in directed graphs arises in transcriptomics where the question is to identify all alternative splicing events present in a sample of mRNAs sequenced by RNA-seq.

**Results:**

We present a new algorithm for enumerating bubbles with length constraints in weighted directed graphs. This is the first polynomial delay algorithm for this problem and we show that in practice, it is faster than previous approaches.

**Conclusion:**

This settles one of the main open questions from Sacomoto et al. (BMC Bioinform 13:5, 2012). Moreover, the new algorithm allows us to deal with larger instances and possibly detect longer alternative splicing events.

## Background

Transcriptomes of model or non model species can now be studied by sequencing, through the use of RNA-seq, a protocol which allows to obtain, from a sample of RNA transcripts, a (large) collection of (short) sequencing reads using Next Generation Sequencing (NGS) technologies [1, 2]. Nowadays, a typical experiment produces 100M reads of 100 nt each. However, the original RNA molecules are longer (typically 500–3,000 nt) and the general computational problem in the area is then to be able to assemble the reads in order to reconstruct the original set of transcripts. This problem is not trivial for mainly two reasons. First, genomes contain repeats that may be longer than the read length. Hence, a read does not necessarily enable to identify unambiguously the locus from which the transcript was produced. Second, each genomic locus may generate several types of transcripts, either because of genomic variants (i.e. there may exist several alleles for a locus) or because of transcriptomic variants (i.e. alternative splicing or alternative transcription start/end may generate several transcripts from a single locus that differ by the inclusion or exclusion of subsequences). Hence, if a read matches a subsequence shared by several alternative transcripts, it is a priori not possible to decide which of these transcripts generated the read.

General purpose transcriptome assemblers [3–5] aim at the general goal of identifying all alternative transcripts from a set of RNA-seq reads, but due to the complexity of the problem several simplifications and approximations are applied, as a result they usually fail to identify infrequent transcripts, tend to report several fragments for each gene, or fuse genes that share repeats. Local transcriptome assemblers [6], on the other hand, aim at a simpler goal as they do not reconstruct full length transcripts. Instead, they focus on reporting all variations, whether genomic (SNPs, indels) or transcriptomic (alternative splicing events). They are much less affected by the issue of repeats, since they focus only on the variable regions. They can afford to be exact and therefore are able to have access to infrequent transcripts. The fundamental idea is that each variant corresponds to a recognizable pattern, called a bubble in the de Bruijn graph (DBG) built from the RNA-seq reads. In practice, only bubbles with specific length constraints are of interest. However, even with this restriction, the number of such bubbles can be exponential in the size of the graph. Therefore, as with other enumeration problems, the best possible algorithm is one spending time polynomial in the input size between the output of two bubbles, i.e. a polynomial delay algorithm.

There were four main algorithmic questions left open in [6]: (i) a polynomial delay algorithm to enumerate bounded length bubbles, (ii) a practical algorithm to retrieve events with a long variable part, (iii) a practical algorithm to retrieve mutually exclusive exons, and (iv) a practical algorithm to deal with complex regions (likely repeat-associated) in DBGs.

In this paper, we provide a solution to the first question and partial one to the second. We introduce the first polynomial delay algorithm to enumerate all bubbles with length constraints in a weighted directed graph. Its complexity in the best theoretical case for general graphs is $O(n(m+n \log n))$ (“[Sec Sec3]”) where $n$ is the number of vertices in the graph, $m$ the number of arcs. In the particular case of de Bruijn graphs, the complexity is $O(n(m+n \log \alpha ))$ (“Dijkstra’s algorithm with different priority queues”) where $\alpha$ is a constant related to the length of the skipped part in an alternative splicing event. In practice, an algorithmic solution in $O(nm\log n)$ (“Comparison with the Kissplice algorithm”) appears to work better on de Bruijn graphs built from such data. We implemented the latter, show that it is more efficient than previous approaches and outline that it allows to discover novel long alternative splicing events. Note that it is out of the scope of this paper to analyze the precision and recall of the algorithm. For that we refer to [6]. Finally, we consider (“A natural generalization”) the enumeration of a structure that is a natural generalization of bubbles.

## De Bruijn graphs and variations in the transcriptome

A DBG is a directed graph $G=(V,A)$ whose vertices $V$ are labeled by words of length $k$ over an alphabet $\Sigma$. An arc in $A$ links a vertex $u$ to a vertex $v$ if the suffix of length $k-1$ of $u$ is equal to the prefix of $v$. The out and the in-degree of any vertex are therefore bounded by the size of the alphabet $\Sigma$. In the case of NGS data, the $k$-mers correspond to all words of length $k$ present in the reads of the input dataset, and only those. In relation to the classical DBG for all possible words of size $k$, the DBG for NGS data may then not be complete. Given two vertices $s$ and $t$ in $G$, an $(s,t)$-path is a path from $s$ to $t$. As defined in [7], by a $(s,t)$-bubble, we mean two vertex-disjoint $(s,t)$-paths. This definition is, of course, not restricted to de Bruijn graphs.

As was shown in [6], variations in a transcriptome (including SNPs, indels, AS events, but not alternative transcription start/end) correspond to recognizable patterns in the DBG that are precisely the $(s,t)$-bubbles. Intuitively, the variable parts correspond to alternative paths and the common parts correspond to the beginning and end points of those paths. More formally, any process generating patterns $awb$ and $aw'b$ in the sequences, with $a,b,w,w' \in \Sigma ^*$, $|a| \ge k, |b|\ge k$ and $w$ and $w'$ not sharing any $k$-mer, creates a $(s,t)$-bubble in the DBG.

**Figure 1:**
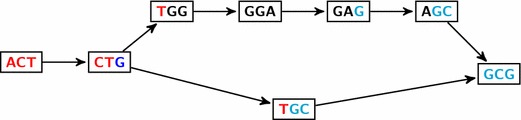
Bubble in DBG. DBG with $k=3$ for the sequences: ACTGGAGCG ($awb$) and ACTGCG ($ab$). The pattern in the sequence generates a $(s,t)$-bubble, from CTG to GCG. In this case, $b=$ GCG and $w=$ GGA have their first letter G in common, so the path corresponding to the junction $ab$ has $k-1-1 = 1$ vertex.

Bubbles can then be classified according to the length of their paths. In the case of SNPs, $$|w|=|w'|=1$$ and each path of the bubble corresponds to the set of $$k$$$$k$$-mers overlapping the variable nucleotide. In the case of genomic indels and most types of AS events (exon skipping, alternative donor/acceptor, intron retention), $$w'$$ is empty and one of the paths corresponds to the *junction* of $$ab$$, i.e. to $$k$$-mers that contain at least one letter of each sequence. Thus the number of vertices of this path in the DBG is predictable: it is at most^a^$$k-1$$. An example is given in Figure 1.

In the special case of mutually exclusive exons, $w'$ is not empty and this restriction on the length of one of the paths does not hold. Additionally, transcriptomes contain repeats, which may also generate bubbles. Repeat-associated bubbles have unpredictable length. In practice, some of these bubbles may have one path with less than $k-1$ nodes. However, they exhibit a high sequence similarity between $w$ and $w'$, a property that can be used to discriminate them, in a post-processing step, from true AS events.

Moreover, in order to optimise the specificity, a lower bound $\beta$ on both paths is imposed [6]. Bubbles with at least one very small path tend to be false positives. Finally, in order to optimize the time performance, and to a lesser extent, the specificity, an upper bound $\alpha$ for the longer path is also imposed [6].

Overall, if we neglect mutually exclusive exons, searching for AS events corresponds to searching for $(s,t)$-bubbles with paths $p_1$ and $p_2$ such that $p_1$ has at most $\alpha$ vertices, $p_2$ at most $k-1$, and both have at least $\beta$ vertices. Increasing the upper bound of $p_2$ to $k$ instead of $k-1$ also captures SNPs.

Given a directed graph $G$ with *non-negative* arc weights $w: E \mapsto \mathbb {Q}_{\ge 0}$, the length of the path $p = (v_0,v_1) \cdots (v_{n-1}, v_n)$ is the sum of the weights of the edges in $p$ and is denoted by $|p|$. The distance, that is the length of the shortest path from $u$ to $v$ is denoted by $d(u,v)$. We extend the definition of bubble given above.

### **Definition 1**

($(s,t,\alpha _1,\alpha _2)$*-bubble*) A $(s,t, \alpha _1,\alpha _2)$*-bubble* in a weighted directed graph is a $(s,t)$-bubble with paths $p_1,p_2$ satisfying $|p_1| \le \alpha _1$ and $|p_2| \le \alpha _2$.

In practice, when dealing with DBGs built from NGS data, in a lossless preprocessing step, all maximal non-branching linear paths of the graph (i.e. all paths containing only vertices with in and out-degree 1) are compressed each into one single vertex, whose label corresponds to the label of the path [i.e. it is the concatenation of the labels of the vertices in the path without the overlapping part(s)]. The resulting graph is the *compressed de Bruijn graph* (cDBG). In the cDBG, the vertices can have labels larger than $k$, but an arc still indicates a suffix-prefix overlap of size $k-1$. Finally, since the only property of a bubble corresponding to an AS event is the constraint on the length of the path, we can disregard the labels from the cDBG and only keep for each vertex its label length. Resulting in a graph with weights in the vertices. Here, however, we consider weights in the arcs. Since this is more standard and, in our case, both alternatives are equivalent, we can transform one into another by splitting vertices or arcs. In this way, searching for bubbles corresponding to AS events in a cDBG can be seen as a particular case of looking for $(s,t, \alpha _1, \alpha _2)$-bubbles satisfying the lower bound $\beta$ in a non-negative weighted directed graph.

Actually, it is not hard to see that the enumeration, for all $s$ and $t$, of $(s,t, \alpha _1,\alpha _2)$-bubbles satisfying the lower bound $\beta$ is NP-hard. Indeed, deciding the existence of at least one $(s,t, \alpha _1,\alpha _2)$-bubble, for some $s$ and $t$, with the lower bound $\beta$ in a weighted directed graph where all the weights are 1 is NP-complete. This follows by a simple reduction from the Hamiltonian $st$-path problem [8]: given a directed graph $G = (V,A)$ and two vertices $s$ and $t$, build the graph $G'$ by adding to $G$ the vertices $s'$ and $t'$, the arcs $(s,s')$ and $(t,t')$, and a new path from $s'$ to $t'$ with exactly $|V|$ vertices. There is a $(x,y,|V|+2,|V|+2)$-bubble, for some $x$ and $y$, satisfying the lower bound $\beta = |V| + 2$ in $G'$ if and only if there is a Hamiltonian path from $s$ to $t$ in $G$.

From now on, we consider the enumeration of all $(s,t,\alpha _1,\alpha _2)$-bubbles (without the lower bound) for a given source (fixed $s$) in a non-negative weighted directed graph $G$ (not restricted to a cDBG). The number of vertices and arcs of $G$ is denoted by $n$ and $m$, respectively.

## An $O(n (m + n \log n))$ delay algorithm

In this section, we present an $O(n (m + n \log n))$ delay algorithm to enumerate, for a fixed source $s$, all $(s,t,\alpha _1,\alpha _2)$-bubbles in a general directed graph $G$ with non-negative weights. In a *polynomial delay* enumeration algorithm, the time elapsed between the output of two solutions is polynomial in the instance size. The pseudocode is shown in Algorithm 1. It is important to stress that this pseudocode uses high-level primitives, e.g. the tests in lines 5, 11 and 19. An efficient implementation for the test in line 11, along with its correctness and analysis, is implicitly given in Lemma 4. This is a central result in this section. For its proof, we need Lemma 2.

Algorithm 1 uses a recursive strategy, inspired by the binary partition method that successively divides the solution space at every call until the considered subspace is a singleton. In order to have a more symmetric structure for the subproblems, we define the notion of a *pair of compatible paths*, which is an object that generalizes the definition of a $(s,t,\alpha _1,\alpha _2)$-bubble. Given two vertices $s_1,s_2 \in V$ and upper bounds $\alpha _1, \alpha _2 \in \mathbb {Q}_{\ge 0}$, the paths $p_1 = s_1 \, \leadsto \, t_1$ and $p_2 = s_2 \, \leadsto \, t_2$ are a *pair of compatible paths* for $s_1$ and $s_2$ if $t_1 = t_2$, $|p_1| \le \alpha _1$, $|p_2| \le \alpha _2$ and the paths are internally vertex-disjoint. Clearly, every $(s,t,\alpha _1,\alpha _2)$-bubble is also a pair of compatible paths for $s_1 = s_2 = s$ and some $t$.

Given a vertex $v$, the set of out-neighbors of $v$ is denoted by $\delta ^+(v)$. Let now $\mathcal {P}_{\alpha _1,\alpha _2}(s_1,s_2,G)$ be the set of all pairs of compatible paths for $s_1$, $s_2$, $\alpha _1$ and $\alpha _2$ in $G$. We have^b^ that:

1 $$\begin{aligned} \mathcal {P}_{\alpha _1, \alpha _2}(s_1,s_2,G) = \mathcal {P}_{\alpha _1, \alpha _2}(s_1,s_2,G^\prime) \bigcup _{v \in \delta ^+(s_2)} (s_2,v) \mathcal {P}_{\alpha _1, \alpha _2^\prime} (s_1,v,G - s_2), \end{aligned}$$

where $\alpha _2' = \alpha _2 - w(s_2,v)$ and $G' = G - \{(s_2,v) | v \in \delta ^+(s_2) \}$. In other words, the set of pairs of compatible paths for $s_1$ and $s_2$ can be partitioned into: $\mathcal {P}_{\alpha _1, \alpha _2'} (s_1,v,G - s_2)$, the sets of pairs of paths containing the arc $(s_2,v)$, for each $v \in \delta ^+(s_2)$; and $\mathcal {P}_{\alpha _1, \alpha _2}(s_1,s_2,G')$, the set of pairs of paths that do not contain any of them. Algorithm 1 implements this recursive partition strategy. The solutions are only output in the leaves of the recursion tree (line 3), where the partition is always a singleton. Moreover, in order to guarantee that every leaf in the recursion tree outputs at least one solution, we have to test if $\mathcal {P}_{\alpha _1, \alpha _2'} (s_1,v,G - s_2)$ (and $\mathcal {P}_{\alpha _1, \alpha _2}(s_1,s_2,G')$) is not empty before making the recursive call (lines 11 and 19).

The correctness of Algorithm 1 follows directly from the relation given in Eq. 1 and the correctness of the tests performed in lines 11 and 19. In the remaining of this section, we describe a possible implementation for the tests, prove its correctness and analyze the time complexity. Finally, we prove that Algorithm 1 has an $O(n(m + n \log n))$ delay.

### **Lemma 2**

*There exists a pair of compatible paths for*$s_1 \ne s_2$* in*$G$* if and only if there exists*$t$* such that*$d(s_1,t) \le \alpha _1$* and*$d(s_2, t) \le \alpha _2$.

### *Proof*

Clearly this is a necessary condition. Let us prove that it is also sufficient. Consider the paths $p_1 = s_1 \, \leadsto \, t$ and $p_2 = s_2 \, \leadsto \, t$, such that $|p_1| \le \alpha _1$ and $|p_2| \le \alpha _2$. Let $t'$ be the first vertex in common between $p_1$ and $p_2$. The sub-paths $p_1' = s_1 \, \leadsto \, t'$ and $p_2' = s_2 \, \leadsto \, t'$ are internally vertex-disjoint, and since the weights are non-negative, they also satisfy $|p_1'| \le |p_1| \le \alpha _1$ and $|p_2'| \le |p_2| \le \alpha _2$. $\square$

Using this lemma, we can test for the existence of a pair of compatible paths for $s_1 \ne s_2$ in $O(m + n \log n)$ time. Indeed, let $T_1$ be a shortest path tree of $G$ rooted in $s_1$ and truncated at distance $\alpha _1$, the same for $T_2$, meaning that, for any vertex $w$ in $T_1$ (resp. $T_2$), the tree path between $s_1$ and $w$ (resp. $s_2$ and $w$) is a shortest one. It is not difficult to prove that the intersection $T_1 \cap T_2$ is not empty if and only if there is a pair of compatible paths for $s_1$ and $s_2$ in $G$. Moreover, each shortest path tree can be computed in $O(m + n\log n)$ time using Dijkstra’s algorithm [8]. Thus, in order to test for the existence of a $(s, t, \alpha _1,\alpha _2)$-bubble for some $t$ in $G$, we can test, for each arc $(s,v)$ outgoing from $s$, the existence of a pair of compatible paths for $s \ne v$ and $v$ in $G$. Since $s$ has at most $n$ out-neighbors, we obtain Lemma 3.

### **Lemma 3**

*The test of line* 5* can be performed in*$O(n (m + n \log n))$.

The test of line 11 could be implemented using the same idea. For each $v \in \delta ^+(u)$, we test for the existence of a pair of compatible paths for, say, $u = s_2$ (the same would apply for $s_1$) and $v$ in $G - u$, that is $v$ is in the subgraph of $G$ obtained by eliminating from $G$ the vertex $u$ and all the arcs incoming to or outgoing from $u$. This would lead to a total cost of $O(n(m+ n \log n))$ for all tests of line 11 in each call. However, this is not enough to achieve an $O(n(m + n \log n))$ delay. In Lemma 4, we present an improved strategy to perform these tests in $O(m+ n \log n)$ total time.

### **Lemma 4**

*The test of line *11*, for all*$v \in \delta ^+(u)$*, can be performed in*$O(m + n \log n)$* total time*.

### *Proof*

Let us assume that $u = s_2$, the case $u = s_1$ is symmetric. From Lemma 2, for each $v \in \delta ^+(u)$, we have that deciding if there exists a pair of compatible paths for $s_1$ and $s_2$ in $G$ that uses $(u,v)$ is equivalent to deciding if there exists $t$ satisfying (i) $d(s_1,t) \le \alpha _1$ and (ii) $d(v,t) \le \alpha _2 - w(u,v)$ in $G - u$.

First, we compute a shortest path tree rooted in $s_1$ for $G-u$. Let $V_{\alpha _1}$ be the set of vertices at a distance at most $\alpha _1$ from $s_1$. We build a graph $G'$ by adding a new vertex $r$ to $G-u$, and for each $y \in V_{\alpha _1}$, we add the arcs $(y,r)$ with weight $w(y,r) = 0$. We claim that there exists $t$ in $G-u$ satisfying conditions (i) and (ii) if and only if $d(v,r) \le \alpha _2 - w(u,v)$ in $G'$. Indeed, if $t$ satisfies (i) we have that the arc $(t,r)$ is in $G'$, so $d(t,r) = 0$. From the triangle inequality and (ii), $d(v,r) \le d(v,t) + d(t,r) = d(v,t) \le \alpha _2 - w(u,v)$. The other direction is trivial.

Finally, we compute a shortest path tree $T_r$ rooted in $r$ for the reverse graph $G'^R$, obtained by reversing the direction of the arcs of $G'$. With $T_r$, we have the distance from any vertex to $r$ in $G'$, i.e. we can answer the query $d(v,r) \le \alpha _2 - w(u,v)$ in constant time. Observe that the construction of $T_r$ depends only on $G-u$, $s_1$ and $\alpha _1$, i.e. $T_r$ is the same for all out-neighbors $v \in \delta ^+(u)$. Thus, we can build $T_r$ only once and use it to answer each test of line 11 in constant time. The cost to build $T_r$ is dominated by the two calls to Dijkstra’s algorithm. Therefore, it takes $O(m + n \log n)$ time to build $T_r$. $\square$

### **Theorem 5**

*Algorithm *1* has*$O(n(m + n \log n))$* delay and uses*$O(m+n)$* space*.

### *Proof*

The height of the recursion tree is bounded by $2n$ since at each call the size of the graph is reduced either by one vertex (lines 13 and 15) or all its out-neighborhood (line 20). After at most $2n$ recursive calls, the graph is empty. Since every leaf of the recursion tree outputs a solution and the distance between two leaves is bounded by $4n$, the delay is $O(n)$ multiplied by the cost per node (call) in the recursion tree. From Lemma 2, line 19 takes $O(m + n \log n)$ time, and from Lemma 4, line 11 takes $O(m + n \log n)$ total time. This leads to an $O(m + n \log n)$ time per call, excluding line 5. Lemma 3 states that the cost for the test in line 5 is $O(n(m + n \log n))$, but this line is executed only once, at the root of the recursion tree. Therefore, the delay is $O(n (m + n \log n))$.

Let us now analyze the memory complexity. We need to store only a single copy of the graph $G$ and for each recursive call we store the difference, i.e. the removed arcs, from the previous graph. The total number of differences stored is at most the size of the graph, since for any path in the recursion tree each arc can be removed only once. Thus, the algorithm uses $O(m+n)$ space. $\square$

## Implementation and experimental results

We now discuss the details necessary for an efficient implementation of Algorithm 1 and the results on two sets of experimental tests. For the first set, our goal is to compare the running time of Dijkstra’s algorithm (for typical DBGs arising from applications) using several priority queue implementations. With the second set, our objective is to compare an implementation of Algorithm 1 to the Kissplice (version 1.8.1) algorithm [6]. For both cases, we retrieved from the *Short Read Archive* (Accession code ERX141791) 14M Illumina 79 bp single-ended reads of a *Drosophila melanogaster* RNA-seq experiment. We then built the DBG for this dataset with $k = 31$ using the Minia algorithm [9, 10]. In order to remove likely sequencing errors, we discarded all $k$-mers that are present less than three times in the dataset. The resulting graph contained 22M $k$-mers, which after compressing all maximal linear paths, corresponded to 600k vertices.

In order to perform a fair comparison with Kissplice, we pre-processed the graph as described in [6]. Namely, we decomposed the underlying undirected graph into biconnected components (BCCs) and compressed all non-branching bubbles with equal path lengths. In the end, after discarding all BCCs with less than four vertices (as they cannot contain a bubble), we obtained 7,113 BCCs, the largest one having 24,977 vertices. This pre-processing is lossless, i.e. every bubble in the original graph is entirely contained in exactly one BCC. In Kissplice, the enumeration is then done in each BCC independently.

### Dijkstra’s algorithm with different priority queues

Dijkstra’s algorithm is an important subroutine of Algorithm 1 that may have a large influence on its running time. Actually, the time complexity of Algorithm 1 can be written as $O(n c(n,m))$, where $c(n,m)$ is the complexity of Dijkstra’s algorithm. There are several variants of this algorithm [8], with different complexities depending on the priority queue used, including binary heaps ($O(m \log n)$) and Fibonacci heaps ($O(m + n \log n)$). In the particular case where all the weights are non-negative integers bounded by $C$, Dijkstra’s algorithm can be implemented using radix heaps ($O(m + n \log C)$) [11]. As stated in “De Bruijn graphs and variations in the transcriptome”, the weights of the de Bruijn graphs considered here are integer, but not necessarily bounded. However, we can remove from the graph all arcs with weights greater than $\alpha _1$ since these are not part of any $(s,t,\alpha _1, \alpha _2)$-bubble. This results in a complexity of $O(m + n \log \alpha _1)$ for Dijkstra’s algorithm.

**Figure 2:**
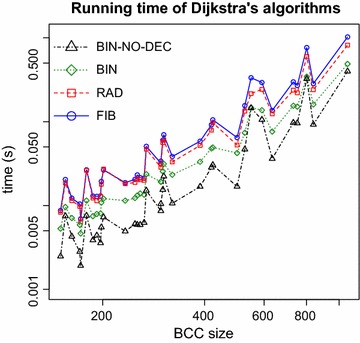
Dijkstra’s algorithms running times. Running times for each version of Dijkstra’s algorithm: using Fibonacci heaps (FIB), using radix heaps (RAD), using binary heaps (BIN) and using binary heaps without the decrease-key operation (BIN-NO-DEC). The tests were done including all BCCs with more than 150 vertices. Both axes are in logarithmic scale. The results for the largest BCC were omitted from the plot to improve the visualization. It took 942.15 s for FIB and 419.84 s for BIN-NO-DEC.

We implemented four versions of Lemma 3 (for deciding whether there exists a $(s,t,\alpha _1, \alpha _2)$-bubble for a given $s$), each using a different version of Dijkstra’s algorithm: with Fibonacci heaps (FIB), with radix heaps (RAD), with binary heaps (BIN) and with binary heaps without decrease-key operation (BIN-NO-DEC). The last version is Dijkstra’s modified in order not to use the decrease-key operation to allow the adoption of a simpler binary heap that does not support such operation [12]. We then ran the four versions, using $\alpha _1 = 1{,}000$ and $\alpha _2 = 2k - 2 = 60$, for each vertex in all the BCCs with more than 150 vertices. The results are shown in Figure 2. Contrary to the theoretical predictions, the versions with the best complexities, FIB and RAD, have the worst results on this type of instances. It is clear that the best version is BIN-NO-DEC, which is at least 2.2 times and at most 4.3 times faster than FIB. One of the factors possibly contributing to a better performance of BIN and BIN-NO-DEC is the fact that cDBGs, as stated in “De Bruijn graphs and variations in the transcriptome”, have bounded degree and are therefore sparse.

### Comparison with the Kissplice algorithm

In this section, we compare Algorithm 1 to the Kissplice (version 1.8.1) enumeration algorithm [6]. To this purpose, we implemented Algorithm 1 using Dijkstra’s algorithm with binary heaps without the decrease-key operation for all shortest paths computation. In this way, the delay of Algorithm 1 becomes $O(nm \log n)$, which is worse than the one using Fibonacci or radix heaps, but is faster in practice. The goal of the Kissplice enumeration is to find all the potential alternative splicing events in a BCC, i.e. to find all $(s,t,\alpha _1,\alpha _2)$-bubbles satisfying also the lower bound constraint (“De Bruijn graphs and variations in the transcriptome”). In order to compare Kissplice to Algorithm 1, we (naively) modified the latter so that, whenever a $(s,t,\alpha _1,\alpha _2)$-bubble is found, we check whether it also satisfies the lower bound constraints and output it only if it does.

**Figure 3:**
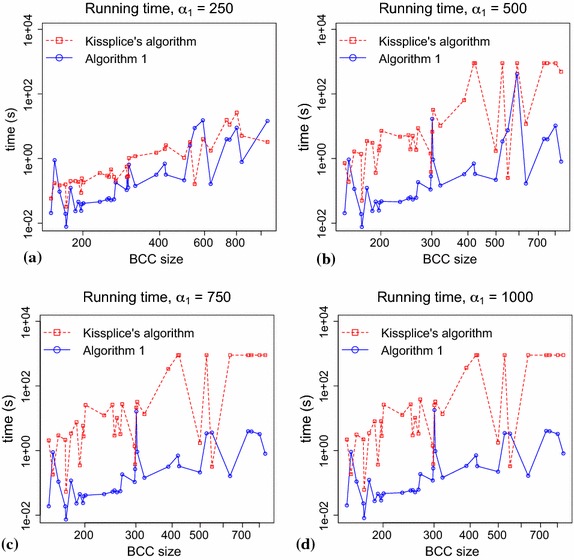
Running times comparison. Running times of Algorithm 1 and of the Kissplice algorithm [6] for all the BCCs with more than 150 vertices. Each graph** a**,** b**,** c** and** d** shows the running time of both algorithms for $\alpha _1 = 250, 500, 750$ and $1{,}000$, respectively. The BCCs where both algorithms reach the timeout were omitted from the plots to improve the visualization. For $\alpha _1 =$ 250, 500, 750 and 1,000 there are 1, 2, 3 and 3 BCCs omitted, respectively.

In Kissplice, the upper bound $\alpha _1$ is an open parameter, $\alpha _2 = k-1$ and the lower bound is $k - 7$. Moreover, there are two stop conditions: either when more than 10,000 $(s,t,\alpha _1,\alpha _2)$-bubbles satisfying the lower bound constraint have been enumerated or a 900 s timeout has been reached. The first stop condition is imposed in Kissplice for specificity reasons, BCCs with more than 10,000 bubbles are likely to contain too many false positives. So, in order to be as close as possible to Kissplice original setup we also use this stop condition in our tests. We ran both Kissplice (version 1.8.1) and the modified Algorithm 1, with the stop conditions, for all 7,113 BCCs, using $\alpha _2 = 60$, a lower bound of $54$ and $\alpha _1 = 250,500,750$ and $1{,}000$. The running times for all BCCs with more than 150 vertices (there are 37) is shown in Figure 3. For the BCCs smaller than 150 vertices, both algorithms have comparable (very small) running times. For instance, with $\alpha _1 = 250$, Kissplice runs in 17.44 s for *all* 7,113 BCCs with less than 150 vertices, while Algorithm 1 runs in 15.26 s.

The plots in Figure 3 show a trend of increasing running times for larger BCCs, but the graphs are not very smooth, i.e. there are some sudden decreases and increases in the running times observed. This is in part due to the fact that the time complexity of Algorithm 1 is output sensitive. The delay of the algorithm is $O(nm \log n)$, but the total time complexity is $O(|\mathcal {B}|nm \log n)$, where $|\mathcal {B}|$ is the number of $(s,t,\alpha _1,\alpha _2)$-bubbles in the graph. The number of bubbles in the graph depends on its internal structure. A large graph does not necessarily have a large number of bubbles, while a small graph may have an exponential number of bubbles. Therefore, the value of $|\mathcal {B}|nm \log n$ can decrease by increasing the size of the graph. A decrease in running time when the size of the graph increases is explained by a smaller number of bubbles in the larger graph.

Concerning now the comparison between the algorithms, as we can see in Figure 3, Algorithm 1 is usually several times faster (keep in mind that the axes are in logarithmic scale) than Kissplice, with larger differences when $\alpha _1$ increases (10 to 1,000 times faster when $\alpha _1 = 1{,}000$). In some instances however, Kissplice is faster than Algorithm 1, but (with only one exception for $\alpha _1 = 250$ and $\alpha _1 = 500$) they correspond either to very small instances or to cases where only 10,000 bubbles were enumerated and the stop condition was met. Moreover, the plots for $\alpha _1 = 750$ and $\alpha _2 = 1{,}000$ seem identical. Actually, the running times are very similar, but not identical, implying that there are few bubbles with upper path larger than $750$ and smaller than $1{,}000$. Finally, using Algorithm 1, the computation finished within 900 s for all but three BCCs, whereas using Kissplice, 11 BCCs remained unfinished after 900 s. The improvement in time therefore enables us to have access to bubbles that could not be enumerated with the previous approach.

Finally, the memory consumption of Kissplice and Algorithm 1 are very similar, since Kissplice also uses memory linear in the size of the graph [6]

### On the usefulness of larger values of $\alpha _1$

In the implementation of Kissplice, the value of $\alpha _1$ was experimentally set to 1,000 due to performance issues, as indeed the algorithm quickly becomes impractical for larger values. On the other hand, the results of “Comparison with the Kissplice algorithm” suggest that Algorithm 1, that is faster than Kissplice, can deal with larger values of $\alpha _1$. From a biological point of view, it is a priori possible to argue that $\alpha _1 = 1{,}000$ is a reasonable choice because 90% of annotated internal exons in *Drosophila* indeed are shorter than 1,000 nt [13]. However, missing the top 10% may have a big impact on downstream analyses of AS events not to mention the possibility that not yet annotated AS events could be enriched in long skipped exons. When studying intron retention, being able to deal with larger values of $\alpha _1$ is critical since introns are notoriously longer than exons. In this section, we give an indication that larger values of $\alpha _1$ indeed produce more results that are biologically relevant. For this, we exploit another RNA-seq dataset, with deeper coverage.

To this purpose, we retrieved 32M RNA-seq reads from human brain and 39M from human liver from the Short Read Archive (accession number ERP000546). Next we built the DBG with $k=31$ for both datasets, then merged and decomposed the DBG into 128 BCCs (containing more than 4 vertices). We ran Algorithm 1 for each BCC with $\alpha _1 = 5{,}000$. There were 114 bubbles with the length of the upper path strictly larger than 1,000 bp. In order to assess if these bubbles were true AS events, we aligned both paths of each bubble to the human reference genome (version hg19) using STAR [14] with default parameters. We found no case where the two paths of a bubble mapped to two distinct genomic locations, which would be a hallmark of a repeat-associated artifactual bubble. We further clustered the bubbles which had the exact same genomic coordinates. The bubbles contained in a cluster correspond to the same AS event, they simply differ by a SNP or an indel in the variable region, which happens frequently for long AS events. Since we are only interested in AS events here, and not in the coupling of genomic variations and AS, we further study only one representative per cluster. We therefore end up with 61 bubbles with unique genomic coordinates.

We classified them according to the number of alignment blocks the two paths generated on the reference and found that out of 61 cases, 19 were intron retentions, 17 were alternative donor or alternative acceptor sites and 25 were exon skipping events (out of which 9 are multiple exons, and 10 are skipped exons combined with an alternative donor or acceptor). In contrast with events smaller than 1,000 nt (a total of 3,540 events), long events are enriched in intron retentions (25 vs. 7%), depleted in exon skippings (44 vs. 54%) and depleted in alternative donors and acceptors (30 vs. 34%). We also compared the genomic locations of the long events with the Ensembl v75 annotation [13]. We found that out of 61 cases, 37 had all their splice sites annotated, while 24 exhibited at least one novel splice site. Out of these 24, 4 contained non-canonical splice sites (i.e. different from GT-AG). In contrast with events smaller than 1,000 nt, the proportion of novel events is larger (39 vs. 14%) and the proportion of non-canonical is similar (18 vs. 17%).

Clearly, the proportions we give in this section are obtained with small numbers and should be interpreted with caution. Furthermore, we rely on STAR for the identification of the splice sites and we cannot exclude that the exact position may be erroneous because of a mapping error. However, we can argue that the vast majority of long bubbles do correspond to true AS events, which were overseen using Kissplice (version 1.8.1). All the annotated AS events predicted by our approach are publicly available^c^.

## A natural generalization

### An intractable case: paths with length constraints

For the sake of theoretical completeness, in this section, we extend the definition of $(s,t,\alpha _1,\alpha _2)$-bubble to the case where the length constraints concern $d$ vertex-disjoint paths, for an arbitrary but fixed $d$. This situation also arises in real data, when more than 2 variants share the same flanking splice sites (for instance for single and double exon skipping), or when a SNP has 3 variants.

#### **Definition 6**

($(s,t,\mathcal{A})$*-d-bubble*) Let $d$ be a natural number and $\mathcal{A} = \{\alpha _1, \ldots , \alpha _d\} \subset \mathbb {Q}_{\ge 0}$. Given a directed weighted graph $G$ and two vertices $s$ and $t$, a $(s,t, \mathcal{A})$-$d$*-bubble* is a set of $d$ pairwise internally vertex-disjoint paths $\{p_1, \ldots p_d\}$, satisfying $p_i = s \, \leadsto \, t$ and $|p_i| \le \alpha _i$, for all $i \in [1,d]$.

Analogously to $(s,t,\alpha _1,\alpha _2)$-bubbles, we can define two variants of the enumeration problem: one seeks all bubbles with a given source ($s$ fixed), while the other identifies all bubbles with a given source and target ($s$ and $t$ fixed). In both cases, the first step is to decide the existence of at least one $(s,t, \mathcal{A})$-$d$-bubble in the graph.

#### **Problem 7**

($(s,t,\mathcal{A})$*-d-bubble decision problem*) Given a non-negatively weighted directed graph $G$, two vertices $s,t$, a set $\mathcal{A} = \{\alpha _1, \ldots , \alpha _d\} \subset \mathbb {Q}_{\ge 0}$ and $d \in \mathbb {N}$, decide if there exists a $(s,t, \mathcal{A})$-$d$-bubble in $G$.

This problem is a generalization of the two-disjoint-paths problem with a min-max objective function, which is NP-complete [15]. More formally, this problem can be stated as follows: given a directed graph $G$ with non-negative weights, two vertices $s,t \in V$, and a maximum length $M$, decide if there exists a pair of vertex-disjoint paths such that the maximum of their lengths is less than $M$. The $(s,t, A)$-$d$-bubble decision problem, with $\mathcal{A} = \{M,M\}$ and $d=2$, is precisely this problem.

#### **Problem 8**

($(s,*,\mathcal{A})$*-d-bubble decision problem*) Given a non-negatively weighted directed graph $G$, a vertex $s$, a set $\mathcal{A} = \{\alpha _1, \ldots , \alpha _d\} \subset \mathbb {Q}_{\ge 0}$ and $d \in \mathbb {N}$, decide if there exists a $(s,t, \mathcal{A})$-$d$-bubble in $G$, for some $t \in V$.

The two-disjoint-path problem with a min-max objective function is NP-complete even for strictly positive weighted graphs. Let us reduce Problem 8 to it. Consider a graph $G$ with strictly positive weights, two vertices $s,t \in V$, and a maximum length $M$. Construct the graph $G'$ by adding an arc with weights $0$ from $s$ to $t$ and use this as input for the $(s,*,\{M,M,0\})$-$3$-bubble decision problem. Since $G$ has strictly positive weights, the only path with length $0$ from $s$ to $t$ in $G'$ is the added arc. Thus, there is a $(s,*,\{M,M,0\})$-$3$-bubble in $G'$ if and only if there are two vertex-disjoint paths in $G$ each with a length $\le M$.

Therefore, the decision problem for fixed $s$ and $t$ (Problem 7) is NP-hard for $d \ge 2$, and for fixed $s$ (Problem 8) is NP-hard for $d \ge 3$. In other words, the only tractable case is the enumeration of $(s,t, \mathcal{A})$-$2$-bubbles with fixed $s$, the one considered in “[Sec Sec3]”.

### A tractable case: paths without length constraints

In the previous section, we showed that a natural generalization of $(s,t,\alpha _1,\alpha _2)$-bubbles to contain more than two vertex-disjoint paths satisfying length constraints leads to an NP-hard enumeration problem. Indeed, even deciding the existence of at least one $(s,t, \mathcal{A})$-$d$-bubble is NP-hard. In this section, we consider a similar generalization for $(s,t)$-bubbles instead of $(s,t,\alpha _1,\alpha _2)$-bubbles, that is, we consider bubbles containing more than two vertex-disjoint paths without any path length constraints. The formal definition is given below.

#### **Definition 9**

($(s,t)$*-d-bubble*) Let $d$ be a natural number. Given a directed graph $G$ and two vertices $s$ and $t$, a $(s,t)$-$d$*-bubble* is a set of $d$ pairwise internally vertex-disjoint paths $\{p_1, \ldots p_d\}$.

Clearly, this definition is a special case of Definition 6: consider a weighted graph $G = (V,E)$ with unitary weights (i.e. an unweighted graph), the $(s,t, \mathcal{A})$-$d$-bubbles with $\alpha _i = |V|$ for $i \in [1,d]$ are precisely the $(s,t)$-$d$-bubbles of $G$. As in “An intractable case: paths with length constraints”, let us first consider the problem of deciding whether a graph contains a $(s,t)$-$d$-bubble for fixed $s$ and $t$.

#### **Problem 10**

($(s,t)$*-d-bubble decision problem*) Given a directed graph $G$ and two vertices $s,t$, decide whether there exists a $(s,t)$-$d$-bubble in $G$.

Contrary to Problem 7, this problem can be decided in polynomial time. Indeed, given a directed graph $G = (V, A)$ and two vertices $s$ and $t$, construct the graph $G' = (V', A')$ by splitting each vertex $v \in V$ in two vertices: an incoming part $v_{in}$ with all the arcs entering $v$, and an outgoing part $v_{out}$ with all the arcs leaving $v$; and add the arc $(v_{in},v_{out})$. More formally, $G'$ is defined as $V' = \{ \{v_{in},v_{out}\} | v \in V \}$ and $A' = \{ (u_{out},v_{in}) | (u,v) \in A \} \cup \{ (v_{in},v_{out}) | v \in V \}$. Now, it is not hard to prove that every set of *arc-disjoint* paths in $G'$ corresponds to a set of vertex-disjoint paths in $G$. Thus, considering $G'$ a network with unitary arc capacities [8], we have that $G$ contains a $(s,t)$-$d$-bubble if and only if $G'$ contains a $(s,t)$-flow $f$ such that $|f| \ge d$. Therefore, using the augmenting path algorithm [8] for the max-flow problem, we can decide if there exists a $(s,t)$-$d$-bubble in $G$ in $O(md)$ time. Actually, using an iterative decomposition of the $(s,t)$-flow $f$ into $(s,t)$-paths, we can explicitly find a $(s,t)$-$d$-bubble in the time bound.

#### **Lemma 11**

*Given a directed graph*$G = (V,A)$* and two vertices *$s,t \in V$*, a*$(s,t)$*-d-bubble in*$G$* can be found in*$O(md)$* time*.

We now consider the problem of enumerating $(s,t)$-$d$-bubbles in $G$ for fixed $s$ and $t$. The reduction from $(s,t)$-$d$-bubbles to $(s,t)$-flows used in the last paragraph may induce us to think that we can enumerate $(s,t)$-$d$-bubbles in $G$ by enumerating $(s,t)$-flows in $G'$, and since there is a polynomial delay algorithm for the latter [16], we would be done. Unfortunately, there is no one-to-one correspondence between $(s,t)$-flows in $G'$ and $(s,t)$-$d$-bubbles in $G$: we can always add a circulation $c$ to a $(s,t)$-flow $f$ to obtain a new $(s,t)$-flow $f'$, but $f$ and $f'$ correspond to the same $(s,t)$-$d$-bubble. In fact, there can be exponentially more $(s,t)$-flows in $G'$ than $(s,t)$-$d$-bubbles in $G$. On the other hand, the strategy used in Algorithm 1 can be adapted to enumerate $(s,t)$-$d$-bubbles.

Similarly to “[Sec Sec3]”, in order to have a more symmetric structure for the subproblems, we define the notion of a *set of compatible paths*, which is an object that generalizes the definition of a $(s,t)$-$d$-bubble. Given a set of sources $S = \{s_1, \ldots , s_d\}$ and a target $t$, a set of paths $P_t = \{p_1, \ldots , p_d\}$ is compatible if $p_i = s_i \, \leadsto \, t$ and they are internally vertex-disjoint. We then focus on the more general problem of enumerating sets of compatible paths. Let $\mathcal {P}(S,t,G)$ be the set of all compatible paths for $S$ and $t$ in $G$. The same partition given in Eq. 1 is also valid for $\mathcal {P}(S,t,G)$. Namely, for any $s \in S$ such that $\delta ^+(s) \ne \emptyset$,

2 $$\begin{aligned} \mathcal {P}(S,t,G) = \mathcal {P}(S,t,G^\prime) \bigcup _{v \in \delta ^+(s)} (s,v) \mathcal {P}(S{\setminus} \{s\} \cup \{v\},t,G - s), \end{aligned}$$

where $G' = G - \{(s,v) | v \in \delta ^+(s)\}$. Now, adding a new source to $G$ with an arc to each vertex in $S$, we can use an augmenting path algorithm to test whether $\mathcal {P}(S,t,G) \ne \emptyset$ in $O(md)$ time. That way, an algorithm implementing the partition scheme of Eq. 2 can enumerate $(s,t)$-$d$-bubbles in $O(n^2md)$ delay, where the bound on the delay holds since each node of the recursion tree costs $O(nmd)$ (at most $n$ emptiness checks are performed) and the height of the tree is bounded by $n$.

#### **Theorem 12**

*Given a directed graph*$G$* and two vertices*$s,t$*, the*$(s,t)$*-d-bubbles in*$G$* can be enumerated in*$O(n^2md)$* delay*.

## Conclusion

We introduced a polynomial delay algorithm which enumerates all bubbles with length constraints in directed graphs. We show that it is faster than previous approaches and therefore enables us to enumerate more bubbles. These additional bubbles correspond to longer AS events, overseen previously but biologically very relevant. Newer versions (from 2.0.0) of Kissplice, source code available on [17], are implemented using Algorithm 1. As shown in [11], by combining radix and Fibonacci heaps in Dijkstra, we can achieve a complexity in $O(n(m + n \sqrt{ \log \alpha _1)})$ for Algorithm 1 in cDGBs. The question whether this can be improved, either by improving Dijkstra’s algorithm (exploiting more properties of a cDBG) or by using a different approach, remains open.

An important question raised in [6], related to this paper, but not considered here is how to deal with complex BCCs. In the tests of “Comparison with the Kissplice algorithm”, the complex BCCs are the ones where the enumeration is not finished, i.e. either there are more than 10,000 bubbles of the 900 s timeout was reached. We have strong indications that their are generated mainly by ancient copies of transposable elements, present in UTRs and intronic regions. Thus, in order to find the AS “trapped” inside complex BCCs we need a proper modeling of the ancient copies of transposable elements present in RNA-seq experiment.

## Endnotes

^a^The size is exactly $k-1$ if $w$ has no common prefix with $b$ and no common suffix with $a$.

^b^The same relation is true using $s_1$ instead of $s_2$.

^c^http://kissplice.prabi.fr/amb2015.
